# The effect of carbamic acid, (1,2,3-thiadiazole-4-ylcarbonyl)-hexyl ester on *Peronophythora litchii* infection, quality and physiology of postharvest litchi fruits

**DOI:** 10.1186/s13065-017-0244-x

**Published:** 2017-02-06

**Authors:** Hai Liu, Guoxing Jing, Yueming Jiang, Fuying Luo, Zaifeng Li

**Affiliations:** 1 School of Chemical Engineering, Xiangtan University, Xiangtan, 411105 People’s Republic of China; 2 College of Food Science and Technology, Modern Biochemistry Experimental Center, Guangdong Ocean University, Zhanjiang, 524088 People’s Republic of China; 3 School of Life Science and Technology, Zhanjiang Normal University, Zhanjiang, 524048 People’s Republic of China; 4 Key Laboratory of Plant Resources Conservation and Sustainable Utilization, South China Botanical Garden, Chinese Academy of Sciences, Guangzhou, 510650 People’s Republic of China

**Keywords:** Litchi fruits, CTE, Postharvest, Quality, Storage

## Abstract

**Background:**

Litchi (*Litchi chinensis* Sonn.) is a subtropical fruit with attractive characteristic of white to creamy semitranslucent flesh and red color in pericap, but it was easily subjected to the infection of *Peronophythora litchii* and lost its market values. Experiments were conducted to understand the effect of [Carbamic acid, (1,2,3-thiadiazole-4-ylcarbonyl)-hexyl ester, CTE] on the growth of *P. litchi* and quality properties in litchi fruits during postharvest storage.

**Results:**

In vitro experiments, CTE with minimum inhibitory concentration (MIC, 5 mg/L) and minimum fungicidal concentration (MFC, 10 mg/L) were against the growth of *P. litchi* for 2 and 4 days, respectively, and SEM results showed that hyphae of *P. litchii* shrank, distorted and collapsed after CTE treatment. In vivo experiments, CTE treatment inhibited the increase of disease incidence, browning index, weight loss and PPO activity in non-*P. litchii*-inoculated fruits, meanwhile the treatment markedly inhibited the decrease of color characteristic (a*, b* and L*), anthocyanin content, phenolic contents, Vc content and POD activity, but TSS content was not significantly influenced during storage. In *P. litchii*-inoculated fruits, all these above mentioned parameters in CTE treated fruits were significantly higher than that in control fruits, but anthocyanin content, Vc, TSS and TA content did not have consistent differences between control and CTE treated fruits at the end of storage.

**Conclusion:**

CTE treatment reduced the disease incidence and browning index of litchi fruits, maintained the fruits quality and, thus, it could be an effective postharvest handling to extend the shelf life of litchi fruits during storage.

**Electronic supplementary material:**

The online version of this article (doi:10.1186/s13065-017-0244-x) contains supplementary material, which is available to authorized users.

## Background

Litchi (*Litchi chinensis* Sonn.) is a subtropical fruit with high commercial value in southern China, it owns the attractive characteristic of white to creamy semitranslucent flesh and red color in pericap [1, 2]. However, the fruits are very susceptible to many diseases and the anthocyanin in pericap degraded quickly during postharvest storage, then the flesh of lithchi deteriorated and lost its market values [3]. The pathogens of *Peronophythora litchii*, is the one of major fungus causing the decay of harvested litchi fruits, resulting in the dispersal of inoculum. The mycelium and oospores of *P. litchii* attacks fruits and causing panicle rot, withering and watery brown spots on fruits, finally sporulating and producing downy white sporangiophores at lately infection [4]. The carboxyl acid amide [CAA, such as dimethomorph (DMM), azoxystrobin (AZB), famoxadone (FMD), metalaxyl (MTL), cymoxanil (CYX) and mancozeb (MCB)] fungicides, were first registered in China for controlling the litchi downy blight, the hypotheses of its action mode included inhibition of phospholipid biosynthesis and interference with cell wall deposition [5, 6]. In addition, the traditional fungicides include mancozeb, cymoxanil and metalaxyl used to control litchi downy blight, and QoI fungicides (such as azoxystrobin, pyraclostrobin, trifloxystrobin and famoxadone) have been used extensively around the globe to control downy mildews [7–9], but the agents resistant isolates have been detected in some regions after using for a long time. Considering pathogen resistance for *P. litchii*, some new alternative means to control the decay of postharvest litchi are required.

Triazoles, like other heterocyclic compounds, are widely used as fungicides for the prevention of many diseases [10]. The mechanism of antifungal activity was triazoles inhibited the demethylation of cytochrome P450 and synthesis of sterol in fungi [11]. Carbamate pesticides are compounds derived from carbamic acid with the chemical structure: R–O–CO–N (CH3)–R′ (the R represents the group of an alcohol, an oxime, or a phenol, and R′ represents a hydrogen or a methyl group). The carbamate owns multisite inhibitors, and could react with thiol groups which presented in the enzymes of fungi [12]. Carbamic acid, (1,2,3-thiadiazole-4-ylcarbonyl)-hexyl ester (CTE), which containing thiadiazole, a carbamate group and a heterocyclic ring, showed a strong fungistatic activity against *Alternaria kikuchiana* and *Gibberella zeae* [13], but the antifungal activities of CTE on the control of litchi postharvest disease was remain undetermined. Based on the potential broad antifungal spectrum of CTE on the diseases [11–13], the objective of this study was conducted to investigate the effect of CTE on the inhibition of *P. litchii* through in vitro and the influence on fruits quality in vivo experiments.

## Methods

### Pathogen

The pathogen of *P. litchii* isolates were preserved in the Laboratory of School of Life Science and Technology, Lingnan Normal University. The fungal pathogen *P. litchii* isolates were cultured for 6 days on potato dextrose agar (PDA) at 28 ± 2 °C, the spore suspension was adjusted to 1 × 10^6^ spores/mL with a hemacytometer and prepared for using.

### In vitro experiments

The fungistatic activity measurement of in vitro experiments was according to the method of Molina Torres [14]. The novel compound of CTE was supported by Ph.D Li, the structure and synthesis scheme were shown in Additional file 1: Figure S2. CTE was dissolved in ethanol (40–50 °C) and added to the PDA culture medium at a temperature of 50–60 °C, the mixtures (with 5, 10 and 20 mg/L CTE, respectively) were poured into Petri dishes of 9 cm in diameter. The solidified plates were inoculated with 6 mm 6-day-old cultures of *P. litchii*, inverted and incubated at 28 ± 2 °C for 144 h. All of the tests were performed in triplicate. The minimum inhibitory concentration (MIC) was the lowest concentration for preventing the pathogen growth for 48 h at 28 ± 2 °C, the lowest concentration that completely inhibited the growth of *P. litchii* after 96 h incubation was represented the minimum fungicidal concentration (MFC). The growth inhibition rates were calculated with the following equation [13]:

$$I = \frac{C - T}{C} \times 100$$

Here, *I* is the growth inhibition rate (%), *C* is the radius (mm) of control plates, and *T* represents the radius (mm) of treatment group.

### Scanning electron microscopy (SEM) for fungal pathogen

4-day-old cultures of *P. litchii* on PDA inoculated with 0, MIC and MFC CTE were prepared for SEM observations [15]. 5 × 5 mm segments from PDA plates were promptly placed in 0.1 M phosphate buffer [pH 7.3, containing 2.5% (v/v) glutaraldehyde] and kept for 24 h at 4 °C for fixation, then washed with distilled water 3 times (20 min each) and dehydrated in an ethanol series (30, 50, 70, and 95%, v/v) for 20 min, finally the samples were dehydrated with absolute ethanol for 45 min and dried in liquid carbon dioxide. After drying, samples were mounted on standard 1/2 in SEM stubs using double-stick adhesive tabs and coated with gold–palladium electroplating (60 s, 1.8 mA, 2.4 kV) in a Polaron SEM Coating System sputter coater. All samples were observed in a FEI Quanta-200 SEM (FEI, USA) operating at 20 kV at 15,000× level of magnification.

### Fruits and pathogen inoculation

Fresh mature fruits of litchi cv. Huaizhi were obtained from an orchard in Zhanjiang, China. Fruits were selected for uniformity of shape, color and free of blemish or disease. The fruits were divided into three groups and infiltrated in a solutions contained sterile distilled water (control), 5 (MIC) and 10 mg/L CTE (MFC) for 2 min. After air-drying, each group of the fruits was divided into two subgroups. One subgroup of control, 5 and 10 mg/L CTE-treated fruits were made 4 equidistant punctures (0.5 mm deep) around the fruit equator with a 1 mm wide sterile nail, and then dipped into the spore suspension of *P. litchii* (1 × 10^6^ spores/mL) for 2 s [3]. The other subgroup fruits were treated under the protocol mentioned above except of being punctured. Then the fruits were packed in 0.03 mm polyethylene bags (250 × 200 mm, 4 bags with 20 fruits per bag), and stored at 25 ± 2 °C and 85–90% relative humidity.

### Disease incidence, browning index and weight loss

The measurements of weight loss, disease incidence and browning index were according to methods of Jing [16]. Weight loss was estimated by testing the weight changes of litchi fruits during storage, and the weight loss rate (%) was calculated by the percentage of initial weight. The signs of fungal existed in the pericap represents the fruits were subjected to infection, disease incidence was recorded the percentage of fungal infection and monitored by 60 fruits in 3 polyethylene bags (0.03 mm thick, 250 × 200 mm) on each pointing time. The browning index means the red color on the pericap of lichi fruits was fade to brown, and the degree of browning index was accessed by the following scale: 0 = no browning; 1 = slight browning; 2 = less than 1/4 browning; 3 = 1/4 to 1/2 browning; and 4 = more than 1/2 browning. The incidence of browning was calculated as:

$${\text{Browning index}} = \frac{{\mathop \sum \nolimits {\text{browning scale }} \times {\text{number of fruits in each class}}}}{{{\text{number of total fruits }} \times {\text{highest browning scale}}}} \times 100$$

Weight loss was estimated by testing the weight changes of litchi fruits during storage, and the weight loss rate (%) was calculated by the percentage of initial weight. All the experiments were made in triplicate.

### Color characteristic

After the calibration of Minolta Chroma Meter CR-400 (Konica Minolta Sensing, Inc, Japan) with the white standard tile, the pericap color characteristics of 6 fruits were determined in the equatorial region [17]. The color values of a* b*, and L* were tracked during storage (a* represents the redness and greenness of litchi fruits, b* represents the yellowness and blueness, L* was used to denote lightness). For these determinations, 6 fruits were used and the experiments were made in triplicate.

### Anthocyanin cotent and phenolic contents

The measurement of anthocyanin cotent was according to the method of Jing [16], 5 g pericarp tissues from 30 fruits were blanched with 200 mL of 0.1 M HCl, the reextraction of recovered tissues were carried out more 2 times until the colorless residue was obtained. The extract solution (5 mL) was diluted in 25 mL of 0.4 M KCl–HCl buffer (pH 1.0), and 25 mL of 0.4 M citric acid-Na_2_HPO_4_ buffer (pH 4.5). The anthocyanin cotent was determined by a photometric assay of using spectrophotometer (UVmini-1240, Shimadzu Corp, Japan) at 510 nm. Total anthocyanin content was expressed as cyanidin-3-glucoside equivalent on a FW basis, and all the experiments were made in triplicate.

The extraction of total phenolic contents was according to the method of Jing [16] with some modification. 5.0 g litchi pericap with 100 mL methanol (containing 0.1 M HCl) was extracted in a shaker for 2 h at 25 °C. The extraction was filtered through a Whatman No. 1 paper (Whatman Inc., Shanghai, China) and the supernatant was used for phenolic contents determination, the content of total phenolic was expressed as gallic acid equivalent on a FW basis.

### Fruits quality parameters

Flesh tissue (20 g) from 6 fruits was homogenized in a grinder and then centrifuged for 20 min at 15,000*g*. The upper phase was collected for the analyses of total soluble solids (TSS), titratable acid (TA) and ascorbic acid (Vc) [16]. The measurement of TSS was determined by using a hand refractometer (J1-3A, Guangdong Scientific Instruments). Titratable acid was determined with 0.1 M NaOH, and ascorbic acid content was determined by 2,6-dichlorophenolindophenol titration. All the experiments were made in triplicate.

### Peroxidase and polyphenol oxidase activities

The determination of POD and PPO activities were according to the method of Jing [16] and Wang [18]. 4.0 g litchi pericap from 30 fruit with potassium phosphate buffer [50 mM, pH 7.0, containing 1% (w/v) polyvinylpyrrolidone] were homogenized in ice-bath and then centrifuged at 10,000×*g* for 15 min at 4 °C, discarding the sediment and the supernatant was the crude enzyme for POD and PPO determination.

Guaiacol as a substrate was used for POD determination, 0.05 mL enzyme extraction was added to the reaction mixture [containing 2.75 mL 50 mM PBS buffer (pH 7.0), 0.1 mL 1% H_2_O_2_ and 0.1 mL 4% guaiacol], the increase of absorbance was recorded for 2 min at 470 nm, the change of 0.01 in absorbance per minute after the addition of enzyme solution was equated to one unit of enzymatic activity. Similarly, oxidation of 4-methylcatechol was used for PPO determination. 100 mL enzyme extraction was mixed with 2.7 mL 200 mM phosphate buffer (pH 7.5) and 200 mL 4-methylcatechol (60 mM) at 25 °C, the change of 0.001 in absorbance at 410 nm per minute was regarded as one unit of enzymatic activity.

### Determination of procyanidin B1, (+)-catechin, (−)-epicatechin and (−)-epicatechin-3-gallate

The measurement of 4 major phenolics according to the method of Jing [16], 1.0 g litchi pericarp with 10 mL of 60% ethanol was extracted in an ultrasonic bath (40 kHZ, SB-5200DTD, Xinzhi Biotech Co., Ningbo, China) at 30 °C for 30 min, then the solution was filtered through a Whatman No. 1 paper (Whatman Inc., Shanghai, China) and evaporated to 2 mL in a rotatory evaporator (RE52AA, Yarong Equipment Co., Shanghai, China), finally the concentrated solution was filtered through 0.45 μm PVD membranes (Shanghai ANPEL Scientific Instruments Co. Ltd., Shanghai, China). The determination of phenolic compounds was separated in a high performance liquid chromatograph (HPLC) (Shimadzu LC-20 AT, Shimadzu Corporation, Japan), coupled with and a SPD-10A UV–VIS detector at 280 nm and a C18 column (218 TP, 250 × 4.6 mm, 5 μm of particle size, Sigma-Aldrich, St. Louis, MO, USA).

15 μL sample was injected and eluted with a gradient system consisting of solvent A (0.1% formic acid) and solvent B (methanol), the mobile phase at a flow rate of 1 mL/min for 45 min, and gradient elution program was as follows: 90% A, from 0 to 5 min; 90–0% A, from 5 to 35 min; 0% A, from 35 to 40 min and 90% A, from 40 to 45 min. Identification of individual phenols was estimated on the basis of their retention times, 4 major phenolic contents were quantified by calibrating against procyanidin B1, (+)-catechin, (−)-epicatechin and (−)-epicatechin-3-gallate standards.

### Data analyses

The experiments were arranged in completely randomized design. Data were presented as the means and standard errors (SE). Data were analyzed by analysis of variance using SPSS version 7.5. Least significant differences (LSD) were used to compare significant effects at the 5% level.

## Results

### In vitro experiments

From Table 1, the results showed that the inhibition on mycelial growth was more effective with the increasing content of CTE, 5-20 mg/L CTE showed totally inhibitory effects on the mycelial growth of *P. litchii* after 2 days culture. After 4 days of culture, only 10 and 20 mg/L CTE treatment totally inhibited the growth of fungal. Therefore, the MIC and MFC of CTE against *P. litchii* were 5 and 10 mg/L, respectively.

**Table 1:** Effect of CTE on the mycelial growth of *P. litchii*

Days of storage (d)	The inhibition of mycelial growth (%)			
	0 mg/L	5 mg/L	10 mg/L	20 mg/L
0	100^a^	100^a^	100^a^	100^a^
2	0^b^	100^a^	100^a^	100^a^
4	0^c^	70.91 ± 11.58^b^	100^a^	100^a^
6	0^c^	74.25 ± 6.13^b^	74.87 ± 7.55^b^	100^a^

Each value is presented as mean ± standard error (n = 3). Different letters of a, b and c are significantly different according to Duncan’s multiple range test at *P* < 0.05

### Scanning electron microscopy

**Fig. 1:**
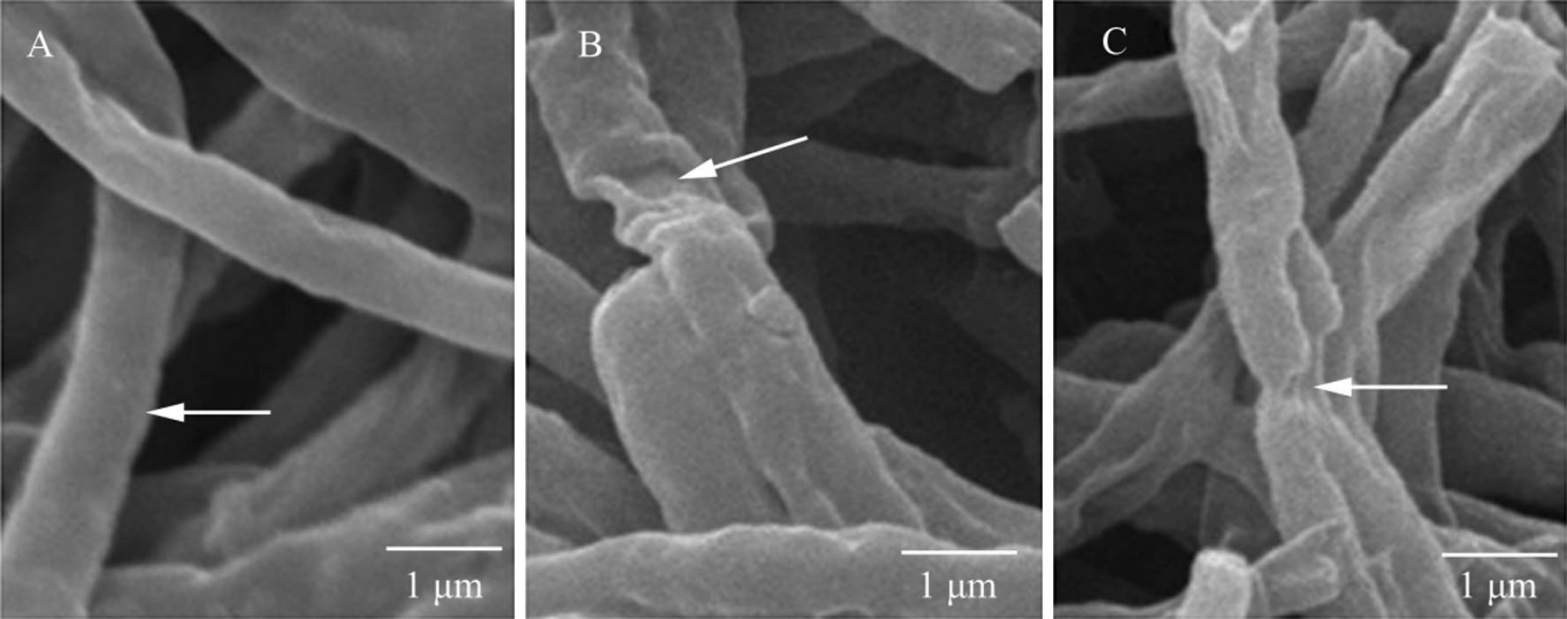
SEM image of *P. litchii*. **A** Mycelia of untreated (control) *P. litchii* with linearly shaped hyphae; **B** *P. litchii* treated with MIC of CTE (the *arrow* refers to the morphologic changes of hyphae after CTE treatment, such as warty surfaces); **C** *P. litchii* treated with MFC of CTE (the *arrow* refers to the morphologic changes of hyphae after CTE treatment, such as collapsed cell)

The growth morphology of *P. litchii* with SEM observation was shown in Fig. 1. The control fungus was regular and homogenous hyphae during culture (Fig. 1A). After 4 days of CTE treatment, the hyphae distorted after MIC treatment and 5 mg/L CTE partly squashed the mycelia (Fig. 1B). Moreover, shrunken and distorted mycelia were observed (Fig. 1C) after treatment with MFC (10 mg/L CTE) for 4 days.

### Disease incidence, pericarp browning and weight loss

**Fig. 2:**
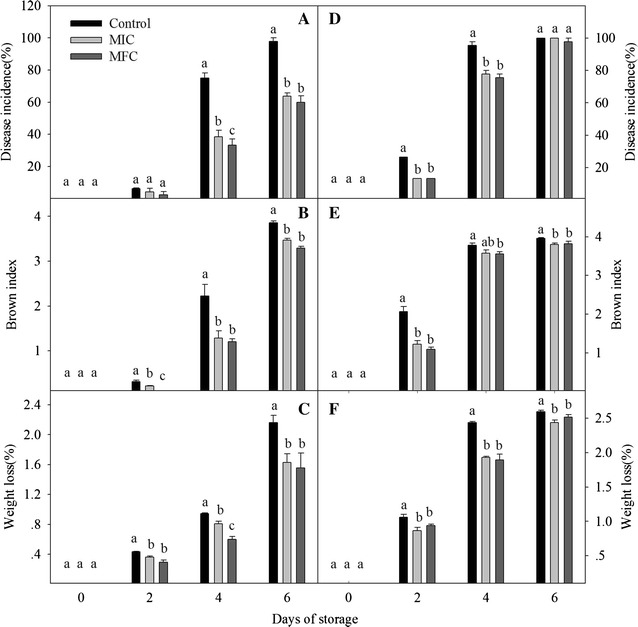
Effect of CTE on the disease incidence, browning index and weight loss in non-*P. litchii*-inoculated (**A**–**C**) and *P. litchii*-inoculated (**D**–**F**) litchi fruit during storage. Each value is presented as mean ± standard error (n = 3). The values in *columns with different letters* indicate a significant (*P* < 0.05) difference between the control, MIC and MFC treated fruits

The incidences of disease, browning and weight loss increased during the storage (Fig. 2). In non-*P. litchii*-inoculated fruits, the disease incidence was low in the early 2 days (Fig. 2A). After 4 days of storage, the disease incidence of control fruits (75.06%) was significantly higher than that in MIC (38.43%) and MFC (33.33%) treated fruits (*P* < 0.05). The control fruits almost completely decayed after 6 days, but the disease incidences of MIC and MFC treated fruits were only 63.79 and 60.00%, respectively. The rotting rate increased rapidly in *P. litchii*-inoculated fruits (Fig. 2D), CTE obviously inhibited the increase of disease incidence, and which were only 77.78 and 75.56% on day 4 in CTE-treated and *P. litchii*-inoculated fruits, but higher rotten rate (95.56%) was found in *P. litchii*-inoculated fruits (*P* < 0.05). Over time, fruits was totally decayed by the inoculation of *P. litchii* at the end of storage.

Similar with the change of disease incidence, the browning index in non-*P. litchii*-inoculated fruits was low (<1) in the first 2 days (Fig. 2B), but it increased rapidly in the subsequent storage. CTE treatment dramatically inhibited the browning of pericap, and which was significantly lower than that in the control fruits (*P* < 0.05). In *P. litchii*-inoculated fruits, the browning index increased rapidly in *P. litchii*-inoculated fruits (Fig. 2E). The browning index in *P. litchii*-inoculated fruits at 2th day was the corresponding level of non-*P. litchii*-inoculated litchi fruits on day 4. Similar with non-*P. litchii*-inoculated fruits, CTE treatment inhibited the increase of browning index in *P. litchii*-inoculated fruits.

In non-*P. litchii*-inoculated fruits, the weight loss of MIC and MFC-treated fruits were 1.63 and 1.56% at 6th day (Fig. 2C), which was significantly lower than that in control fruits (2.16%). In *P. litchii*-inoculated fruits, the weight loss of *P. litchii*-inoculated fruits increased rapidly and reached to 1.06% on day 2 (Fig. 2F), which was the equivalent values of non-*P. litchii*-inoculated fruits at 4th day. As the same, CTE treatment obviously inhibited the increase of weight loss in *P. litchii*-inoculated fruits in the first 4 days of storage (*P* < 0.05), but there was no significant difference between the CTE-treated and control fruits on day 6.

### Color characteristic

**Fig. 3:**
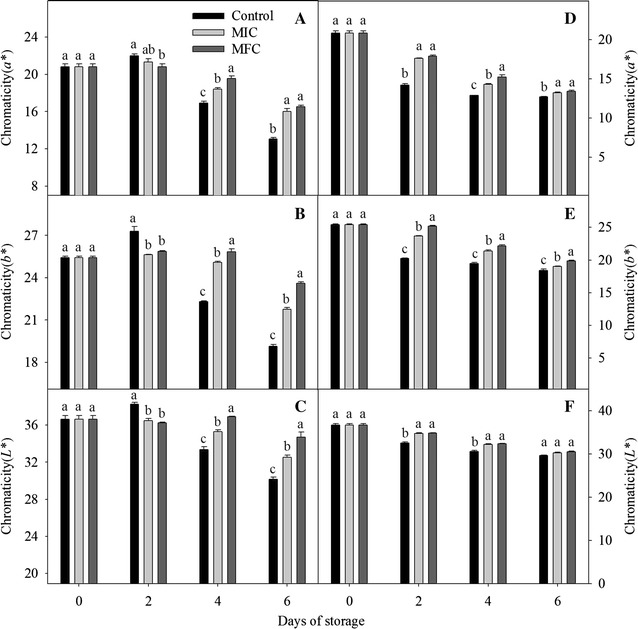
Effect of CTE on the color characteristic of a*, b* and L* in non-*P. litchii*-inoculated (**A**–**C**) and *P. litchii*-inoculated (**D**–**F**) litchi fruit during storage. Each value is presented as mean ± standard error (n = 3). The values in *columns with different letters* indicate a significant (*P* < 0.05) difference between the control, MIC and MFC treated fruits

As shown in Fig. 3, significant differences in color parameters (a*, b* and L*) were observed between control and CTE-treated fruits. In non-*P. litchii*-inoculated fruits, the a*, b* and L* of control fruits increased and exhibited higher values than CTE-treated fruits in the first 2 days. But in the subsequent storage, the decrease of a*, b* and L* were inhibited by CTE treatment, and the values were significantly higher than that in control fruits (*P* < 0.05). The a*, b* and L* of *P. litchii*-inoculated fruits decreased in all storage days, CTE treatment obviously delayed the decrease of a*, b* and L* values, but the L* value was not exhibited significant difference between the CTE-treated and control fruits at 6th day.

### Anthocyanin and phenolics contents

**Fig. 4:**
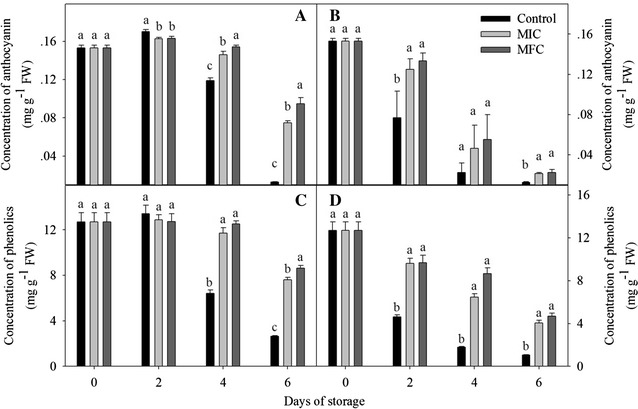
Effect of CTE on concentration of anthocyanin and phenolics in non-*P. litchii*-inoculated (**A**, **C**) and *P. litchii*-inoculated (**B**, **D**) litchi fruit during storage. Each value is presented as mean ± standard error (n = 3). The values in *columns with different letters* indicate a significant (*P* < 0.05) difference between the control, MIC and MFC treated fruits

As shown in Fig. 4A, average anthocyanin contents in non-*P. litchii*-inoculated fruits rose initially and then declined during storage. Anthocyanin of control fruits increased to highest content (0.17 mg/g FW) at 2th day and decreased in subsequent storage. CTE-treatment inhibited the decrease of anthocyanin contents after 2 days of storage, and the content was significantly higher than that in control fruits (*P* < 0.05). The anthocyanin content in *P. litchii*-inoculated fruits decreased during the storage, CTE-treated and *P. litchii*-inoculated fruits showed higher anthocyanin content than control fruits during storage (Fig. 4B).

Similar with the change of anthocyanin contents, the phenolics contents in non-*P. litchii*-inoculated fruits was slightly increased in the first 2 days and then reduced in subsequent storage (Fig. 4C). The contents in control fruits was higher in first 2 days, but which was obviously decreased and significantly lower than that in treated fruits in the rest storage (*P* < 0.05). The phenolics contents in *P. litchii*-inoculated fruits decreased in all storage time and which in CTE treatment groups was significantly higher than control fruits (*P* < 0.05, Fig. 4D).

### Content of Vc, titratable acid and total soluble solid

**Fig. 5:**
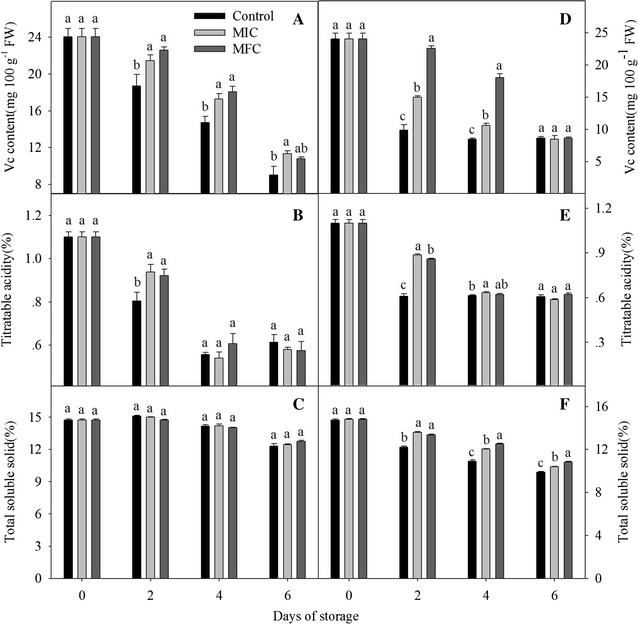
Effect of CTE on content of Vc, titratable acid and total soluble solid in non-*P. litchii*-inoculated (**A**–**C**) and *P. litchii*-inoculated (**D**–**F**) litchi fruit during storage. Each value is presented as mean ± standard error (n = 3). The values in *columns with different letters* indicate a significant (*P* < 0.05) difference between the control, MIC and MFC treated fruits

As shown in Fig. 5A, D, Vc content in the pulp of non-*P. litchii*-inoculated and *P. litchii*-inoculated fruits decreased during the storage. In non-*P. litchii*-inoculated fruits, the Vc content of CTE-treated fruits were significantly higher than that in control fruits (*P* < 0.05). Compared with control fruits in *P. litchii*-inoculated group, the Vc content in CTE-treated and *P. litchii*-inoculated fruits were much higher in the first 4 days of storage (*P* < 0.05).

The content of TA decreased during the storage (Fig. 5B, E). In non-*P. litchii*-inoculated fruits, the TA content of CTE-treated fruits was significantly higher than control in first 2 days of storage, but there were no significant difference between the control and CTE-treated fruits in subsequent storage. TA content of fruits decreased sharply after *P. litchii*-inoculation, MIC treatment inhibited the decrease but no difference was observed between the control and CTE-treated fruits at the end of storage.

As shown in Fig. 5C, TSS in the pulp of non-*P. litchii*-inoculated increased initially and then declined during storage. The content in control increased to the highest content (13.3%) on day 2, which was higher than that in CTE-treated fruits. Then the TSS content decreased but no significant difference was observed between the control and CTE-treated fruits in subsequent storage. In *P. litchii*-inoculated fruits, TSS content decreased continuously and the CTE treatment obviously inhibited the decline during the storage (*P* < 0.05).

### POD and PPO activities

**Fig. 6:**
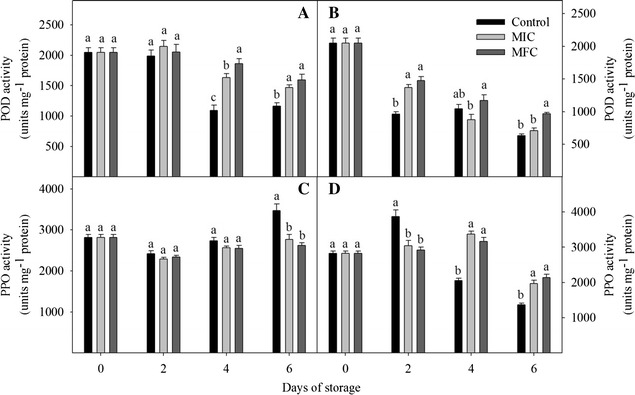
Effect of CTE on activities of peroxidase (POD) and polyphenol oxidase (PPO) in non-*P. litchii*-inoculated (**A**, **C**) and *P. litchii*-inoculated (**B**, **D**) litchi fruit during storage. Each value is presented as mean ± standard error (n = 3). The values in *columns with different letters* indicate a significant (*P* < 0.05) difference between the control, MIC and MFC treated fruits

The change of POD and PPO activities were shown in Fig. 6. After CTE treatment, POD activity of non-*P. litchii*-inoculated fruits increased in first 2 days of storage and then decreased in subsequent storage time, POD activities in CTE treatment groups were significantly higher than control at 4th and 6th day. In *P. litchii*-inoculated fruits, the POD activity decreased during all storage times, CTE treatment slowed down the reduction of enzyme activity and the POD activity in MFC treatment was significantly higher than that in control at 2th and 6th day.

In non-*P. litchii*-inoculated fruits, the PPO activity decreased in first 2 days and then increased slightly in subsequent storage times. There was no significant difference between the control and CTE-treated fruits in first 4 days, but the activity in control was significantly higher than that in CTE treated groups at the end of storage. In first 2 days of *P. litchii*-inoculated fruits, PPO activity in control increased in all days, and which was significantly higher than that in treated fruits. Then the enzyme activity decreased in control but which in CTE treated groups increased till 4th day. Moreover, the activities in CTE-treated fruits were significantly higher than control fruits at the end of storage.

### Contents of procyanidin B1, (+)-catechin, (−)-epicatechin and (−)-epicatechin-3-gallate

Four phenolic compounds of procyanidin B1, (+)-catechin, (−)-epicatechin and (−)-epicatechin-3-gallate were determinated in the litchi pericarp (Table 2). The content of procyanidin B1 decreased during storage, but CTE treatment inhibited the decrease of procyanidin B1 content. Moreover, procyanidin B1 was not detected at 6th day in non-*P. litchii*-inoculated fruits. The decrease was obviously in *P. litchii*-inoculated fruits, procyanidin B1 was not detected after 2 days in control, but CTE treatment inhibited the decrease and procyanidin B1 was not detected at the end of storage.

**Table 2:** Effect of CTE on the content of procyanidin B1, (+)-catechin, (−)-epicatechin and (−)-epicatechin-3-gallate in non-*P. litchii*-inoculated (a, c) and *P. litchii*-inoculated (b, d) litchi fruit during storage

Phenolic compounds (mg/g FW)	Treatments concentration	Non-*P. litchii*-inoculated fruits				*P. litchii*-inoculated fruits			
		0 day	2 days	4 days	6 days	0 day	2 days	4 days	6 days
Procyanidin B1	0	0.085 ± 0.002^a^	0.083 ± 0.002^a^	0.033 ± 0.00^c^	–^c^	0.085 ± 0.002^a^	–^c^	–^c^	–^a^
	MIC	0.085 ± 0.002^a^	0.074 ± 0.002^b^	0.065 ± 0.003^b^	0.035 ± 0.001^b^	0.085 ± 0.002^a^	0.048 ± 0.002^b^	0.036 ± 0.001^b^	–^a^
	MFC	0.085 ± 0.002^a^	0.074 ± 0.004^b^	0.07 ± 0.002^a^	0.054 ± 0.002^a^	0.085 ± 0.002^a^	0.058 ± 0.002^a^	0.039 ± 0.001^a^	–^a^
(+)-Catechin	0	0.92 ± 0.041^a^	1.01 ± 0.07^a^	0.45 ± 0.02^c^	0.31 ± 0.01^c^	0.92 ± 0.041^a^	0.48 ± 0.02^b^	0.22 ± 0.01^c^	–^c^
	MIC	0.92 ± 0.041^a^	0.96 ± 0.03^ab^	0.67 ± 0.03^b^	0.43 ± 0.02^b^	0.92 ± 0.041^a^	0.74 ± 0.03^a^	0.58 ± 0.02^b^	0.26 ± 0.01^b^
	MFC	0.92 ± 0.041^a^	0.93 ± 0.03^b^	0.76 ± 0.04^a^	0.53 ± 0.03^a^	0.92 ± 0.041^a^	0.77 ± 0.02^a^	0.63 ± 0.03^a^	0.41 ± 0.02^a^
(−)-Epicatechin	0	1.87 ± 0.034^a^	1.89 ± 0.053^a^	0.94 ± 0.024^c^	0.64 ± 0.02^c^	1.87 ± 0.034^a^	0.73 ± 0.02^c^	0.61 ± 0.02^c^	0.54 ± 0.02^c^
	MIC	1.87 ± 0.034^a^	1.91 ± 0.062^a^	1.47 ± 0.05^b^	0.87 ± 0.03^b^	1.87 ± 0.034^a^	1.24 ± 0.05^b^	0.98 ± 0.03^b^	0.67 ± 0.02^b^
	MFC	1.87 ± 0.034^a^	1.86 ± 0.046^a^	1.64 ± 0.032^a^	1.05 ± 0.08^a^	1.87 ± 0.034^a^	1.45 ± 0.06^a^	1.13 ± 0.05^a^	0.74 ± 0.03^a^
(−)-Epicatechin-3-gallate	0	1.67 ± 0.083^a^	1.70 ± 0.07^ab^	1.27 ± 0.05^b^	0.94 ± 0.04^c^	1.67 ± 0.083^a^	0.88 ± 0.04^c^	0.72 ± 0.03^c^	0.70 ± 0.03^b^
	MIC	1.67 ± 0.083^a^	1.65 ± 0.04^b^	1.58 ± 0.03^a^	1.15 ± 0.05^b^	1.67 ± 0.083^a^	1.27 ± 0.06^b^	0.93 ± 0.04^b^	0.81 ± 0.06^b^
	MFC	1.67 ± 0.083^a^	1.73 ± 0.07^a^	1.64 ± 0.03^a^	1.31 ± 0.06^a^	1.67 ± 0.083^a^	1.48 ± 0.08^a^	1.21 ± 0.07^a^	0.96 ± 0.05^a^

Each value is presented as mean ± standard error (n = 3). The values in columns with different letters indicate a significant (*P* < 0.05) difference between the control, MIC and MFC treated fruits

Mostly, (+)-catechin, (−)-epicatechin and (−)-epicatechin-3-gallate increased to highest contents at 2th day, and then decreased in non-*P. litchii*-inoculated fruits. *P. litchii*-inoculation accelerated the degradation of phenolics, and the contents decreased during all storage time. Generally, the contents in CTE treated fruits were higher than control in non-*P. litchii*-inoculated and *P. litchii*-inoculated fruits.

## Discussion

The morphology difference between the control and CTE-treated *P. litchii* hyphae was demonstrated by SEM images (Fig. 1). After MIC of CTE treatment, hyphae of *P. litchii* shrank and formed a rough surface. Moreover, mycelium were distorted and collapsed exposed with MFC concentration. The SEM results indicated CTE treatment might disrupt the plasmalemma of *P. litchii*, increase the leakage of small molecular substances and ions. Accordingly, the growth of *P. litchii* was affected after CTE treatment.

Chemical control is the primary method for controlling litchi posthavest diseases [19, 20]. It had proved that oxalic acid [21] and apple polyphenols [2] could controll pericarp browning and extend the shelf life of harvested litchi fruits. Jing [16] demonstrated that pyrogallol could deley the increase of pericarp browning and fruits decay, and pyrogallol treatment could be beneficial for postharvest litchi fruits storage (4 or 25 °C). Jiang [22] reported that NaHSO_3_ combined with HCl could delay the degradation of anthocyanin on the litchi pericap. Yi [3] found ATP treatment accelerated the defense activity of litchi infected by *P. litchii*, and had a positive influence on disease resistance of litchi fruits. Triazoles act as surface protectants and enter plant tissues as systemic fungicides, but it was site specific inhibitors and easier to develop resistance for fungi [16]. The carboxyl acid amide (CAA) fungicides were extensively used for against different downy mildews, the antifungal mechanism was that multisite inhibitors in CAA could react with the thiol groups presented in the enzymes of fungi, but the CAA-resistant isolates of *Plasmopara viticola* and *Pseudoperonospora cubensis* have been detected in some European regions, South Korea, Israel, the United States and China after long usage [23]. Alkyl N-(1,2,3-thiadiazole-4-carbonyl) carbamates are new classes of lead compounds for controlling plant fungal diseases, their activities depend on the length of the alkyl chain with the optimal length of 6–11 carbons. The novel compounds of CTE containing the structure of thiadiazole, CAA and 6 carbon chains, carbamate group and heterocyclic ring of CTE exhibited a broad and systemic antifungal activity, but with less potential to develop resistance. The linkage atom of oxygen and the length of the alkyl chain were also very critical for fungicidal activity in CTE [13], Li [13] reported that CTE showed a strong fungistatic activity against *A. kikuchiana* at 50 mg/L. In the present study, CTE incorporated into growth media (PDA) were found to inhibit mycelia growth of *P. litchii*, the results of in vitro experiments showed that 5 and 10 mg/L CTE inhibited the growth of *P. litchii* for 2 and 4 days (Table 1), respectively. And in vivo experiments, CTE treatment obviously inhibited the increase of disease incidence and browning index in CTE-treated and *P. litchii*-inoculated fruits (Fig. 2), the results of in vitro and in vivo experiments showed that CTE could be an effective antifungal agent for litchi postharvest storage. As the same, CTE treatment obviously delayed the decrease of a*, b* and L* (Fig. 3), meanwhile the anthocyanin content of CTE-treated fruits was higher than that in control fruits pericap (Fig. 4). The results showed that CTE treatment could preserve the red color in pericap and maintain litchi market values with longer storage, but no obviously difference was observed between the control and CTE treated fruits in *P. litchii*-inoculated groups at 6th day. The compounds of triazoles and CAA might be the multisite inhibitors for antifungal activity, the 6 carbons in the alkyl chain are optimal for the fungicidal activity, so CTE could not be easier for fungi to develop resistance with a different mode of action [13].

Total soluble solids, titratable acidity, Vc content and weight loss often reflect taste quality in flesh. Previous reports demonstrated that chemical treatments not only reducing pericarp browning and fruits rotten, but also preserving functional and sensory quality of litchi fruits [19, 20]. Jing reported that the content of TSS and TA tended to decrease over time, with little effect of storage temperature (4 or 25 °C). Compared with control fruits, average values of TSS were higher in the fruits treated with pyrogallol at different levels, whereas the two groups had similar levels of TA. In the present study, the TSS content was not influenced after CTE treatment during storage, but the TSS content in CTE-treated fruits was higher than that in the control at the end of storage. Similarly, TA content in CTE-treated fruits were higher than that in control fruits, but no obviously differences was observed between the control and treated fruits after 2 days of storage. TSS content decreased continuously and CTE slowed the decline in *P. litchii*-inoculated fruits, whereas no difference was observed in TA content between the control and CTE-treated fruits at the end of storage. The reducing Vc content significantly contributed to the antioxidant activity, which could protect plant tissues against different biotic and abiotic stresses [24]. In our study, the Vc content of CTE-treated fruits were significantly higher than that in control fruits, these differences became more evident with increasing concentration of CTE, and the higher Vc content reflected higher quality in fruits after CTE treatment. Loss of water typically reduced in litchi fruits quality, Jiang and Fu [25] found that higher respiration of litchi fruit packed in polyethylene bags accelerated the weight loss of fruits. Riederer [1] found the functional structure of stomata was lacked in the mature state of litchi fruits, and which resulted in the development of the protective structure against water loss and the adverse impact of the abiotic and biotic environment. In our study, the weight loss of MIC and MFC-treated fruits were significantly lower than that in control fruits. 5 mg/L and 10 mg/L CTE reduced the water loss of litchi, it was proposed that CTE maintained the integrity of water loss barriers and decreased the probability of disease infection in litchi pericap.

Zhang [26] reported that anthocyanins in litchi pericarp was degraded to anthocyanidin during storage, then anthocyanidin was degraded after the action of POD and PPO. In our study, although the POD activity decreased in *P. litchii*-inoculated fruits, the CTE treatment could largely maintain the activity during storage. The results demonstrated that oxidative stress species such as H_2_O_2_ was removed by the higher POD activity, so the CTE treatment prolonged the storage of litchi fruits. Jiang [27] reported that PPO could react with its substrates of phenolic compounds and then result in the browning of litchi fruit, PPO activity was decreased in CTE treated fruits and might reduce the reaction opportunities of PPO with phenolics, so there was significant higher contents of total phenolics and 4 major phenolic compounds (procyanidin B1, (+)-catechin, (−)-epicatechin and (−)-epicatechin-3-gallate) in CTE treated fruits. In short, CTE treatment increased the POD activity for eliminating oxidative damage in litchi fruits, meanwhile the reducing PPO activity weakened the oxidization of phenolics and alleviated the browning of litchi fruits.

## Conclusion

In conclusion, CTE exhibited antifungal activity against *P. litchii* in vitro experiment, with MIC and MFC values of 5 and 10 mg/L, respectively. CTE treatment inhibited the increase of disease incidence, browning index, weight loss and PPO activity in non-*P. litchii*-inoculated fruits, meanwhile the treatment markedly inhibited the decrease of color characteristic (a*, b* and L*), anthocyanin content, phenolic contents, Vc content and POD activity. In *P. litchii*-inoculated fruits, there were no consistent differences in these parameters between control and *P. litchii*-inoculated fruits at the end of storage. Overall, CTE helped to maintain overall quality attributes and it was suggested that the application of CTE could be beneficial in the browning and decay control of litchi fruits.

## Additional file

**Additional file 1: Figure S1.** Identification of 4 phenolic compounds in HPLC chromatogram, a=procyanidin B1; b=catechin; c=(−)- epicatechin and d=(−)- epicatechin −3-gallate, there retention time were 16.3, 18.4, 20.9 and 22.3 min, respectively. **Figure S2.** The disease incidence of non-*P. litchii*-inoculated fruits in Control, MIC and MFC treatments after 6 days of storage. **Figure S3.** The disease incidence of *P. litchii*-inoculated fruits in Control, MIC and MFC treatments after 6 days of storage.
