# Supplementary material for: The effect of carbamic acid, (1,2,3-thiadiazole-4-ylcarbonyl)-hexyl ester on Peronophythora litchii infection, quality and physiology of postharvest litchi fruits
Source: Chem Cent J. 2017 Feb 6;11:14. doi: 10.1186/s13065-017-0244-x (PMC5293705; doi:10.1186/s13065-017-0244-x)

**Figure captions:**

**Fig. S1.** Identification of 4 phenolic compounds in HPLC chromatogram, a=procyanidin B1; b=catechin; c=(−)- epicatechin and d=(−)- epicatechin −3-gallate, there retention time were 16.3, 18.4, 20.9 and 22.3 min, respectively.

Fig. S2. The disease incidence of non-*P. litchii*-inoculated fruits in Control, MIC and MFC treatments after 6 days of storage

Fig. S3. The disease incidence of *P. litchii*-inoculated fruits in Control, MIC and MFC treatments after 6 days of storage

**Fig. S1.**


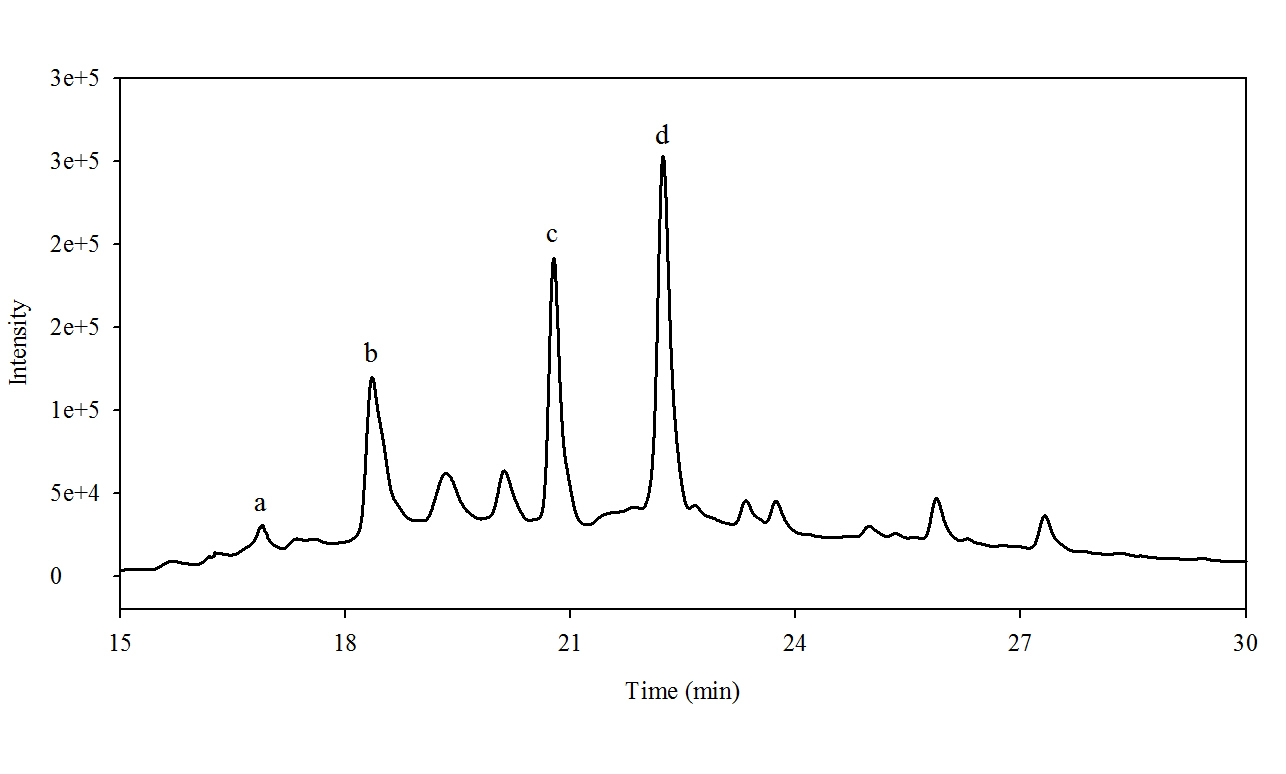


**Fig. S2.**


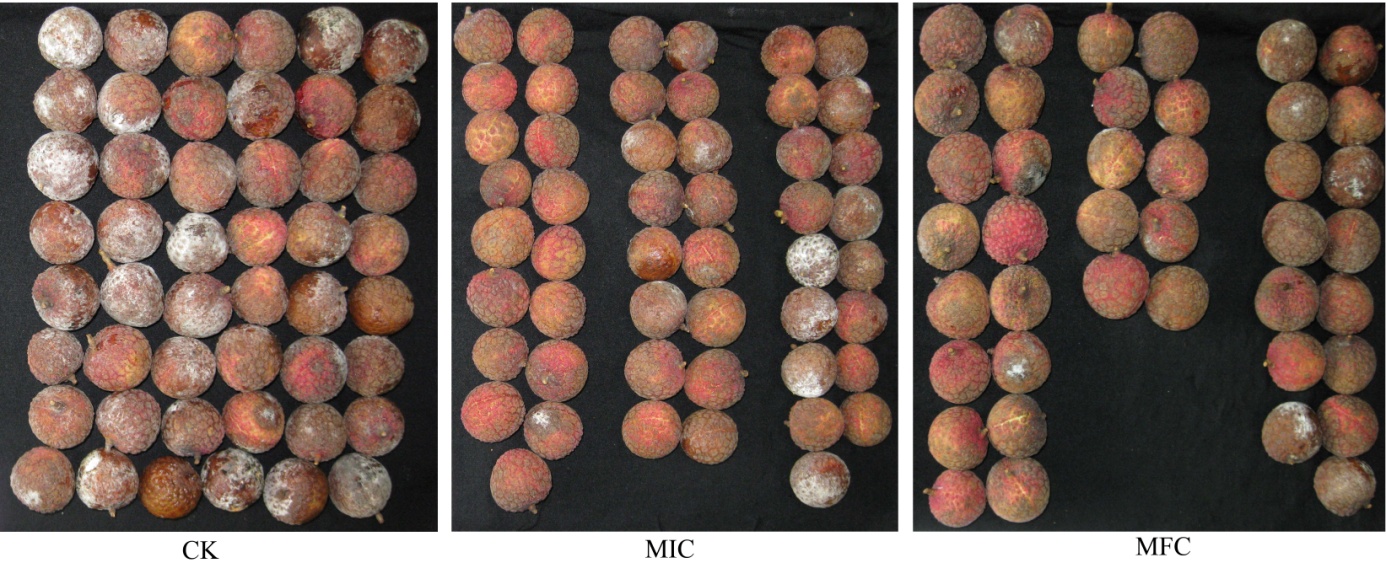


**Fig. S3.**


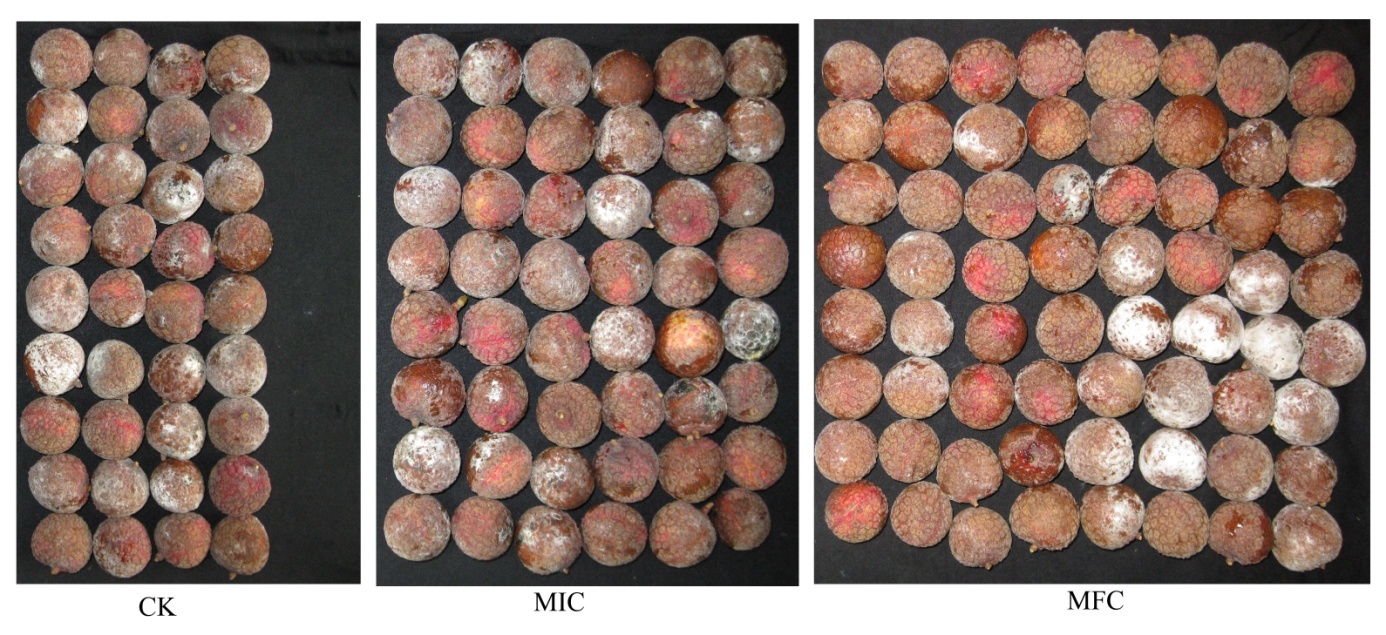

Supplement: Supplementary file 1 — Additional file 1: Figure S1. Identification of 4 phenolic compounds in HPLC chromatogram, a=procyanidin B1; b=catechin; c=(−)- epicatechin and d=(−)- epicatechin −3-gallate, there retention time were 16.3, 18.4, 20.9 and 22.3 min, respectively. Figure S2. The disease incidence of non-P. litchii-inoculated fruits in Control, MIC and MFC treatments after 6 days of storage. Figure S3. The disease incidence of P. litchii-inoculated fruits in Control, MIC and MFC treatments after 6 days of storage. [file 13065_2017_244_MOESM1_ESM.doc]
